# The Characterization of Silicone-Tungsten-Based Composites as Flexible Gamma-Ray Shields

**DOI:** 10.3390/ma14205970

**Published:** 2021-10-11

**Authors:** Jie Wang, Haoyu Zhou, Yong Gao, Yupeng Xie, Jing Zhang, Yaocheng Hu, Dengwang Wang, Zhiming You, Sheng Wang, Haipeng Li, Guoming Liu, Aijun Mi

**Affiliations:** 1 Shaanxi Engineering Research Center of Advanced Nuclear Energy, Shaanxi Key Laboratory of Advanced Nuclear Energy and Technology, School of Nuclear Science and Technology, School of Energy and Power Engineering, Xi’an Jiaotong University, Xi’an 710049, China; wangjie1@xjtu.edu.cn (J.W.); gaoyong1108@stu.xjtu.edu.cn (Y.G.); xieyupeng@stu.xjtu.edu.cn (Y.X.); zhangjing1108@stu.xjtu.edu.cn (J.Z.); hyc1997@stu.xjtu.edu.cn (Y.H.); wdw21s@stu.xjtu.edu.cn (D.W.); youzm19960311@stu.xjtu.edu.cn (Z.Y.); lihaipeng@xjtu.edu.cn (H.L.); 2 Department of Engineering Physics, Tsinghua University, Beijing 100084, China; zhouhy20@mails.tsinghua.edu.cn; 3 China Nuclear Power Engineering Co., Ltd., Beijing 100840, China; liugma@cnpe.cc (G.L.); miaj@cnpe.cc (A.M.)

**Keywords:** gamma-ray shielding, radiation, soft nuclear robot, nuclear safety, nano TiO_2_

## Abstract

Robots are very essential for modern nuclear power plants to monitor equipment conditions and eliminate accidents, allowing one to reduce the radiations on personnel. As a novel robot, a soft robot with the advantages of more degrees of freedom and abilities of continuously bending and twisting has been proposed and developed for applications in nuclear power industry. Considering the radiation and high-temperature environment, the overall performance improvement of the flexible materials used in the soft nuclear robot, such as the tensile property and gamma-ray shielding property, is an important issue, which should be paid attention. Here, a flexible gamma-ray shielding material silicone-W-based composites were initially doped with nano titanium oxide and prepared, with the composition of 20 silicone-(80-*x*) W-(*x*) TiO_2_, where *x* varied from 0.1 to 2.0 wt.%. Structural investigations on SEM and EDS were performed to confirm the structure of the prepared composites and prove that all the chemicals were included in the compositions. Moreover, the tensile property of the composites at 25, 100, and 150 °C were investigated to study the effect of working temperature on the flexibility of the compositions. The attenuation characteristics including the linear attenuation coefficients and mass attenuation coefficients of the prepared silicone-W or silicone-W-TiO_2_-based composites with respect to gamma ray were investigated. The stability of the silicone–tungsten-TiO_2_-based composite at high temperature was studied for the first time. In addition, the influence of nano TiO_2_ additive on the property’s variation of silicone-W-based composites was initially studied. The comparison of the properties such as the tensile elongation, thermal stability, and gamma-ray shielding of the synthesized silicone-W and silicone-W-TiO_2_ composites showed that the addition of nano TiO_2_ powders could be useful to develop novel gamma-ray-shielding materials for radiation protection of soft robots or other applications for which soft gamma-ray-shielding materials are needed.

## 1. Introduction

Various kinds of robots for applications such as monitoring equipment conditions [1,2,3,4], radiation measurements [5,6], etc., are needed in nuclear environments to replace manual work and protect personal safety [7,8,9,10].

A wall-climbing-robot with autonomous driving properties was proposed by Daewon Kim et al. [5] to measure radiation in nuclear power plants (NPPs). A crawler robot combined with Compton cameras [6] was used for radiation imaging and recognizing radioactive contamination in Fukushima Daiichi NPP. In order to navigate in radioactive environments and search for sources in nuclear accidents, obstacle avoidance robots [1] were developed based on fuzzy logic.

Considering the maintenance, dismantling, and decommissioning of nuclear facilities, a tracked robot, RICA [11], was used for radiological inspection, data collection, and sampling to locate and measure the radiation level. Moreover, the ground mobile robot mentioned above and the aerial robot were also adopted for radiological data collection, analysis, and transmission to ensure the security of nuclear sites.

Compared with traditional rigid robots, soft robots feature various advantages, including better flexibility and operability, safe human–computer interaction, low collision damage, and strong adaptability to the environment, etc. In the past few years, various kinds of soft robots [12], such as the soft robot hand [13,14,15], bioinspired soft robotics [16,17], etc., have been proposed and studied, which can be used in fields such as healthcare, human–machine interfacing, electronic, etc. Nowadays, soft robotics have come into being with amplified flexibility, strength, and rigidity to mimic biomimetic motions and perform tasks in uncertain environments [18]. Martin Held et al. [17] synthesized and characterized a biodegradable and stretchable elastomer in soft electronic platforms, which can be applied in biofriendly packaging and healthcare. A wearable robot based on a shape-memory alloy [19] was also proposed for assisting the wrist motion of patients.

After the Fukushima nuclear disaster, many robots have been developed to enter areas with high level radioactivity. Besides aerial and ground rigid robots, soft robots were also proposed for sampling, radiation detection, and measurement [18]. In light of high radioactivity, potential high temperature, and high pressure [11,20,21] in those areas, the materials of the soft robots were required to be flexible and irradiation- and high-temperature-resistant.

Because of the special properties of nanoscale materials, it can be used for the improvement of functional characteristics of composites, such as the mechanical property. A research by A.S. Abouhaswa et al. [22] indicated that the addition of titanium oxide was beneficial for improving its performance in absorbing and shielding gamma radiation. Iman. M. Nikbin et al. [23] found that with the increase in mass ratio of nano titanium oxide, the compressive strength of the heavy concrete composites increased by 44.9%. Ozge Kilicoglu et al. [24] prepared bioactive glasses containing titanium oxide additive and found that those with 2.5 mol % titanium oxide additive revealed the highest gamma-ray attenuation performance. Y. Al-Hadeethi et al. [25] also reported that bioactive glasses with titanium oxide doping manifested improved the gamma-ray and neutron-shielding properties.

Therefore, in order to improve the overall performance of the gamma-ray-shielding silicone-W-based composites, such as the flexibility and high-temperature resistances, nano titanium oxide was doped in it. In this paper, the influence of titanium oxide additive on the property’s variation of silicone-W-TiO_2_-based composites was initially studied and analyzed.

## 2. Materials and Methods

### 2.1. Materials Preparation

The chemical compositions of silicone-W and silicone-W-TiO_2_ gamma-ray-shielding composite samples investigated are presented in Table 1. The 20 silicone-(80-*x*) W-(*x*) TiO_2_ (with *x* varying from 0.1, 0.5, 1.0, and 2.0 wt.%) composites were prepared and characterized as shielding materials against gamma radiation. The purity of nano TiO_2_ with grain size of 5–10 nm (Xfnano Company, Nanjing, China) and W powders (Xinda Company, Xingtai, China) with average particle diameter of approximately 10 μm were 99.3% and 99.9%, respectively. The silicone used in this paper contained group A (platinum cata-lyst-H_2_PtCl_6_ 6H_2_O and Divinyl tetramethyl disiloxane-C_8_H_18_OSi, Tengda Guijiao Science and Technology Ltd., Dongguan, China) and group B (Methyl hydrosilicone oil-(CH_3_)_3_SiO[(CH_3_) (H) SiO]_n_Si(CH_3_)_3_), Tengda Guijiao Science and Technology Ltd., Dongguan, China).

Firstly, silicone group A and group B with the same mass ratio of 10 wt.% were stirred and mixed uniformly. Then, the weighed tungsten and nano titanium dioxide powders were stirred and mixed well before being added into the silicone. Secondly, the mixed composites were stirred for 15 min. Thirdly, the prepared mixtures were stored in a vacuum chamber with a pressure of 133 Pa for 40 min to remove air bubbles in the samples. Finally, the samples were taken out from the vacuum chamber for complete solidification at room temperature and atmospheric pressure for about 36 h.

### 2.2. Characterization Method

The tensile properties of the samples at room temperature and high temperature were tested by the Instron MicroTester 5848 (Instron Limited, Boston, MA, USA) and the Zwick Roell Z005 mechanical property tester (Zwick Roell, Ulm, Germany), respectively. Two types of dumbbell-shaped samples were prepared for the room-temperature (type 4, ISO 37:2005) test and high-temperature (type 1, ISO 37:2005) test with a displacement rate of 30 mm/min. For the tensile property tests at high temperature, all samples and the text device needed to be preheated in advance until the temperature was stable at investigated temperature for 20 min. When changing the sample, it was necessary to keep the temperature in the text device at a preset value for 5 min before the next test.

The compositions of the composites were characterized by Shimadzu 6100 X-ray diffraction (XRD, Shimadzu, Kyoto, Japan). Surface morphology and element distribution were carried out by a JEOL 7800 F scanning electron microscope (SEM, Tokyo, Japan) and an energy dispersive spectrometer (EDS, JEOL Ltd., Tokyo, Japan). The mass variation and calorimetric effects of the composites were analyzed by a STA 449 C thermogravimetric and differential scanning calorimetry (TG-DSC, NETZSCH, Selb, Germany). The TG and DSC results were calibrated by the baseline of empty crucibles to eliminate system errors. Thermal degradations were characterized with a heating rate of 10 °C/min from 30 to 800 °C under N_2_ atmosphere (30 mL/min).

**Figure 1:**
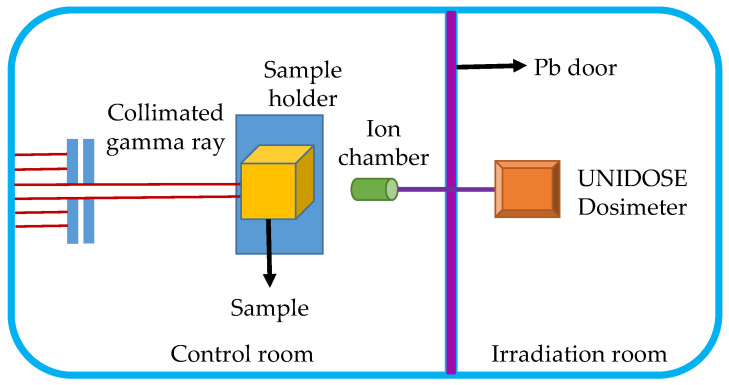
The diagram of gamma-ray radiation transmission experimental setup.

Measurements of gamma-ray linear attenuation coefficients were performed by ^60^ Co source with an intensity of about 3.7 × 10^15^ Bq (Northwest Nuclear Technology Institute, Xi’an, China). The diagram of the experimental setup is shown in Figure 1. The control room and irradiation room were separated by a Pb door. Two gamma-ray energies (1.173 and 1.332 MeV) were released during the tests with a dose rate of 429.4 Gy h^−1^. The gamma-ray intensity was tested by Farmer 30,013 ionization chamber, and the ionizing current formed during tests were recorded by UNIDOSE Dosimeter (PTW, Freiburg, Germany). The collimated gamma-ray intensities right before the sample and after transmitting the sample were recorded. Then, linear attenuation coefficients and mass attenuation coefficients could be calculated.

## 3. Results

### 3.1. Surface Morphologies

**Figure 2:**
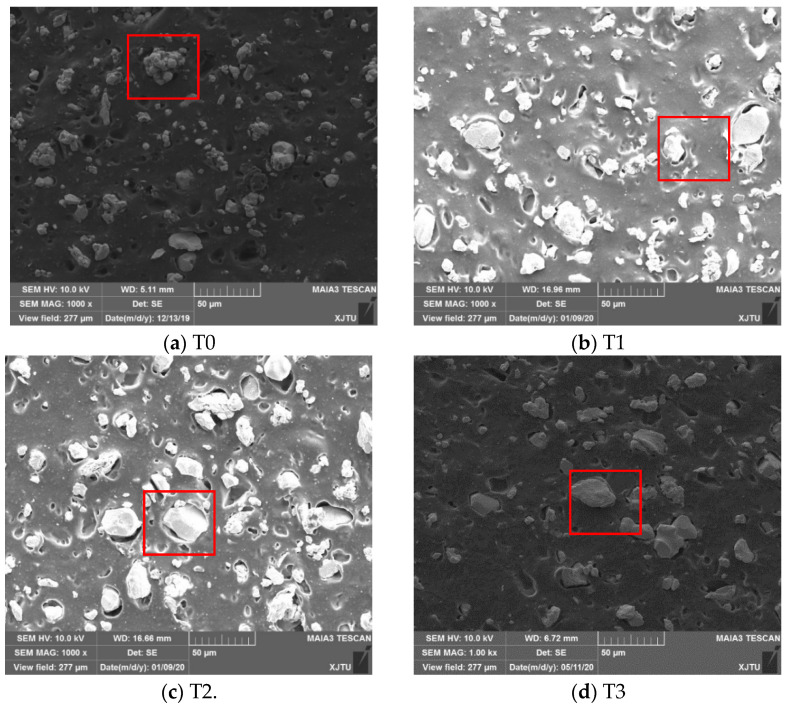

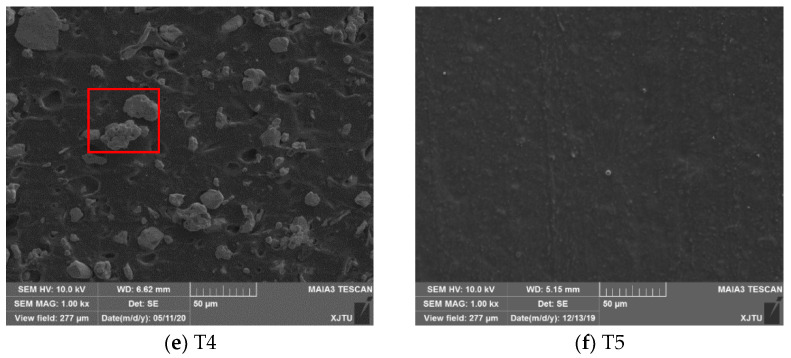
Surface morphologies for samples (**a**) T0, (**b**) T1, (**c**) T2, (**d**) T3, (**e**) T4, and (**f**) T5.

**Figure 3:**
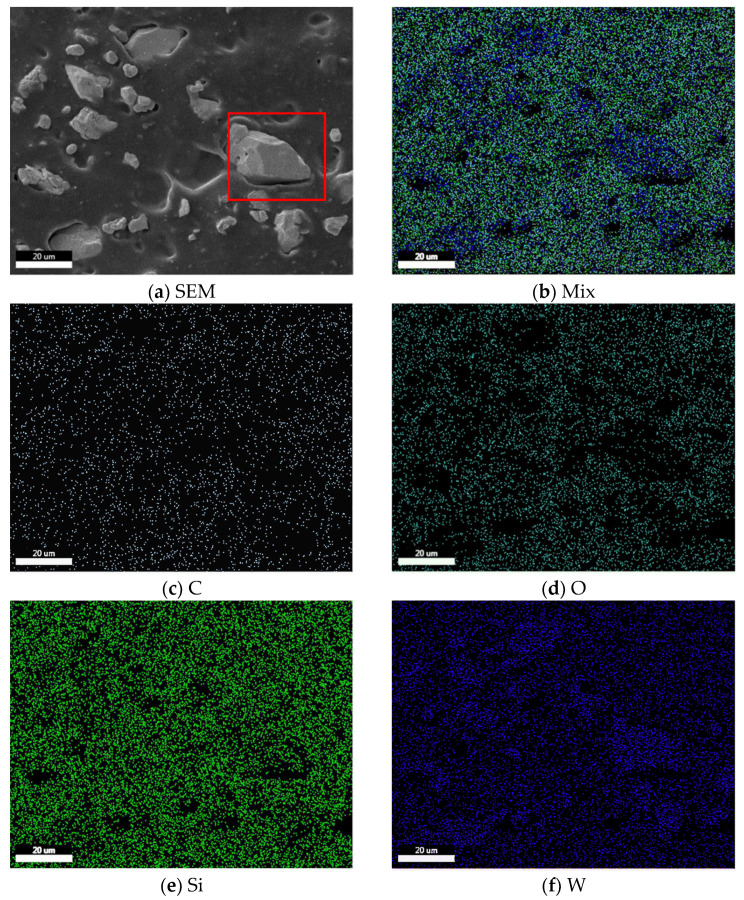

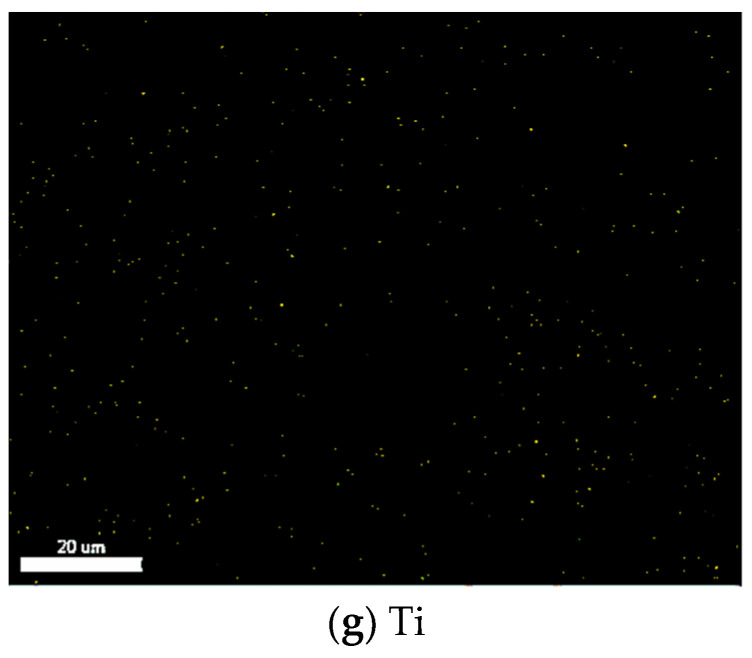
(**a**) Selected representative surface morphology image for sample T2 and its elemental mapping of the elements: (**b**) mix (**c**) C, (**d**) O, (**e**) Si, (**f**) W, and (**g**) Ti.

The surface morphologies of silicone sample T5, silicone-W-based composite sample T0, silicone-W-TiO_2_-based composite samples T1, T2, T3, and T4 are shown in Figure 2. Taking the elemental mapping of sample T2 for example, the blocky-shaped particles in the red rectangle of Figure 3a was tungsten powders (see Figure 3f). It also indicated that the blocky-shaped particles in the red rectangles of Figure 2a–e were also tungsten powders. Therefore, the surface morphology of silicone sample T5 (Figure 2f) without tungsten and TiO_2_ powders was much smoother than that of silicone-W-based or silicone-W-TiO_2_-based composite sample T0, T1, T2, T3, and T4. As shown in Figure 3b–g, the C, O, Si, W, and Ti elements were basically uniformly distributed in the composites.

### 3.2. Tensile Property

**Figure 4:**
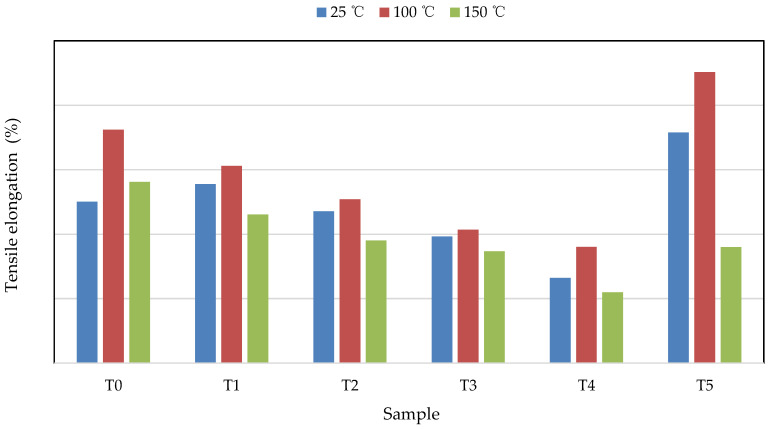
The average tensile elongations of samples T0, T1, T2, T3, T4, and T5 at different test temperatures of 25 (blue), 100 (red), and 150 °C (green).

The average tensile properties of the prepared silicone-based matrix composite samples T0, T1, T2, T3, T4, and T5 at 25, 100, and 150 °C were shown in Figure 4. The tensile elongations of the pure silicone sample T5 at 25 and 100 °C were the highest among all the investigated samples, while, at the temperature of 150 °C, the tensile elongation of sample T1 was the highest.

As the mass ratios of TiO_2_ increased from 0.1 to 2.0 wt.%, the tensile elongation of the silicone-W-TiO_2_-based composites at 100 and 150 °C decreased gradually. Compared with the tensile elongation of silicone-W-based composite T0 at 25 °C, when the mass ratios of TiO_2_ increased from 0.1 to 2.0 wt.%, that of the silicone-W-TiO_2_-based composites increased by about 10.96% and then decreased by about 5.91–47.19%. This indicates that the addition of 0.1 wt.% nano TiO_2_ powder (sample T1) is conducive to improving the tensile elongation at 25 °C. When the mass ratio of nano TiO_2_ powder increased from 0.1 to 2 wt.%, the tensile elongations of silicone-W-TiO_2_-based composites at 100 and 150 °C decreased significantly by about 15.48–50.22% and 18.02–60.90%, respectively, compared to that of sample T0.

### 3.3. Thermogravimetric and Differential Scanning Calorimetry (TG-DSC)

**Figure 5:**
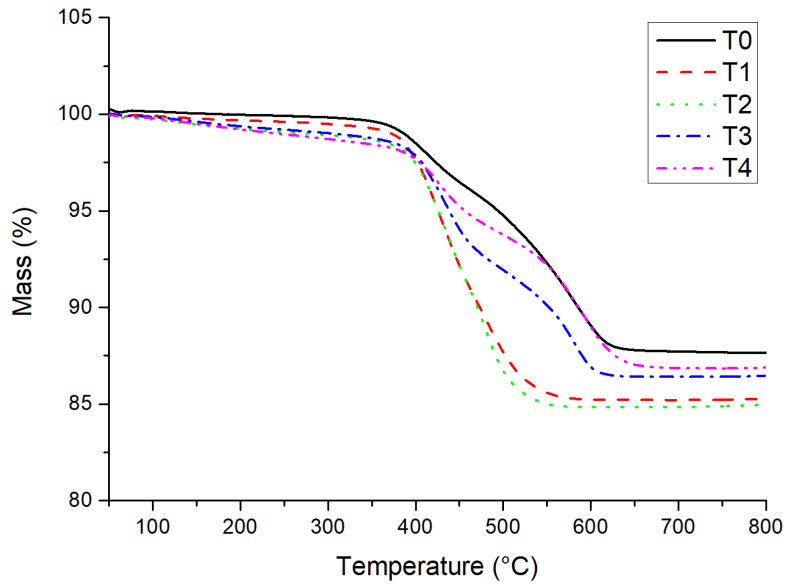
Thermogravimetric curves for samples T0, T1, T2, T3 and T4.

Figure 5 illustrates the TG curves of sample T0 consisting of 20 wt.% silicone and 80 wt.% tungsten, T1 (W/TiO_2_ = 79.9:0.1), T2 (W/TiO_2_ = 79.5:0.5), T3 (W/TiO_2_ = 79.0:1.0), and T4 (W/TiO_2_ = 78.0:2.0) composed of 20 wt.% silicone, tungsten, and nano TiO_2_ with different weight ratios. In the test temperature range from 400 to 600 °C, the steep slopes of the TG curves of these five samples appeared. When the temperature exceeded 600 °C, the TG curves flattened out.

As indicated in Table 2, when the working temperature was less than 300 °C, the mass losses of these five samples were around 0.17–1.29%. In the test temperature range from 500 to 600 °C, the rapid mass losses (5.71–4.72%) of samples T0 and T4 were documented, which may be attributed to the decomposition of silicone, such as the interaction between hydroxyl groups (-OHs) and oxygen atoms in polysiloxane backbone, the scission of polysiloxane backbone [26,27]. For samples T1, T2, and T3, the rapid mass losses (5.92–10.71%) were recorded. The total mass losses of the silicone-W-based composites doped with 0.1–2.0 wt.% nano titanium oxide were 13.15–15.17%, which increased slightly by about 0.80–2.81% compared to that of the silicone-W-based composite sample T0.

**Figure 6:**
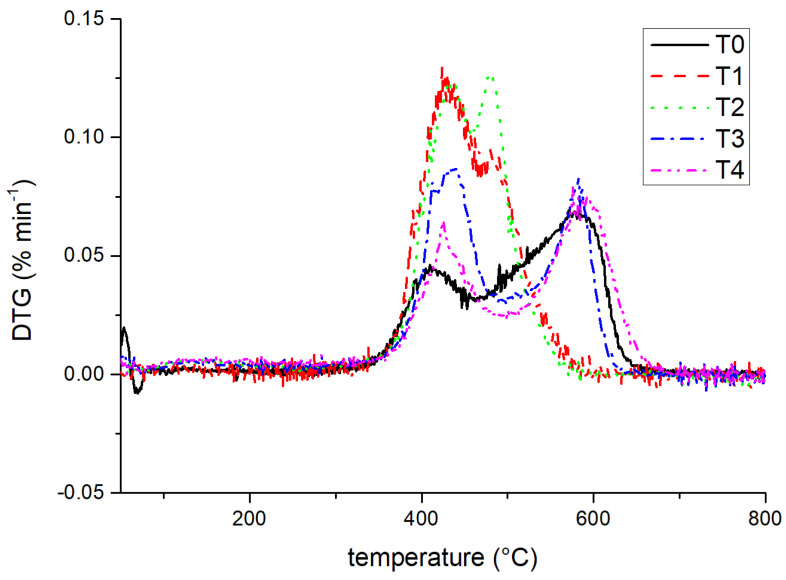
Derived thermogravimetric (DTG) curves for samples T0, T1, T2, T3 and T4.

Figure 6 shows that silicone-W-based composite sample T0 had three endothermic peaks each at 63.8, 409.8, and 571.8 °C, respectively. For samples T1, T2, T3, and T4, two endothermic peaks appeared in the derived thermogravimetric curves (see Figure 6). The first major decomposition temperatures of T1, T2, T3, and T4 were 430.0, 430.3, 436.2, and 425.3 °C with the weight losses of about 5.25%, 5.50%, 4.80%, and 3.51%, respectively. The second endothermic peaks of T1, T2, T3, and T4 appeared at 479.9, 479.2, 582.2, and 576.1 °C, with the weight losses of about 10.59%, 11.09%, 11.91%, and 9.30%, respectively. It demonstrated the very close decomposition temperatures of silicone-W-TiO_2_-based composite samples T1 and T2 with the nano TiO_2_ weight ratios of 0.1 and 0.5 wt.%, respectively, while, when the nano TiO_2_ weight ratios were 1.0 and 2.0 wt.%, the second decomposition temperatures of T3 and T4 were close to the third decomposition temperature of T0.

### 3.4. X-ray Diffraction (XRD)

**Figure 7:**
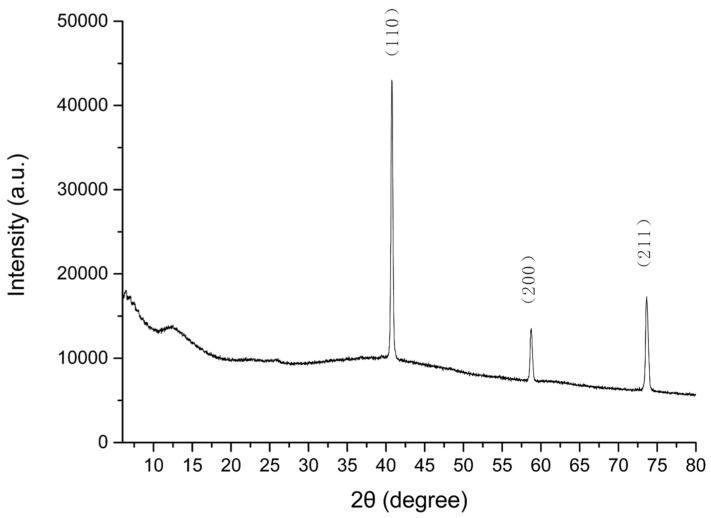
XRD patterns of sample T2 (20.0 wt.% silicone, 79.5 wt.% W, and 0.5 wt.% TiO_2_).

A case study on the phase constitution results of sample T1 is shown in Figure 7. The peaks at 2θ = 40.74°, 58.74°, and 73.65° in the XRD pattern were ascribed to (110), (200) and (211) of tungsten, respectively [28,29,30]. The phase of nano TiO_2_ was not detected, which may be attributed to its low weight concentration.

### 3.5. Gamma-Ray-Shielding Property

The gamma ray with the initial intensity “*I*_0_” traverses a composite with a thickness “*x*”, having a residual intensity “*I*” of the primary photons. The relation of “*I*_0_”, “*x*”, and “*I*” can be given by Equation (1) below:

*I* = *I*_0_ exp(*−μx*) (1)

where *μ* is the total linear attenuation coefficient of the composite. The mass attenuation coefficient with the unit of cm^2^/g is equal to *μ* divided by the composite density “*ρ*”.

As shown in Table 3, when nano TiO_2_ powders with mass ratios of 0.1–2.0 wt.% were doped in the silicone-W-based composite, the linear attenuation coefficients of T1, T2, T3, and T4 decreased slightly by about 1.5–5.3%, compared to that of sample T0. In addition, the mass attenuation coefficient of T1, T2, T3, and T4 reduced slightly by about 1.1–4.8% compared to that of sample T0. This demonstrates that the addition of nano TiO_2_ powders has a slight effect on the gamma-ray-shielding property of the silicone-W-based composite.

The half-value layer (HVL) of the material is defined as follows:

HVL = (ln2)/*μ* = 0.693/*μ* (2)

which means the thickness reducing the intensity of a beam to one-half its initial intensity. The mean free path (MFP) is the average distance that a beam traverses an object before an interaction, which is shown below:

MFP = 1/*μ* (3)

As reported by Mansour Almurayshid et al., the linear attenuation of the composites containing ethylene vinyl acetate (EVA) polymer and Si or SiC or B_4_C at the gamma-ray energy of 0.662 MeV is around 0.9 cm^−1^ [31]. The linear attenuation of the silicone–tungsten based composites here is higher than that of EVA-Si based composites, which may ascribe to the high concentration of tungsten used in our composites. The research from Ahmed Khalaf Mheemeed et al. indicates that nitrile-butadiene rubber–lead mixtures have linear attenuation coefficients of 0.212–0.493 cm^−1^ at 0.662 MeV with the lead weight concentrations between 5 and 75 wt.% [32]. The linear attenuation coefficient difference may be caused by that the atomic number of W being lower than that of Pb. Higher atomic number may contribute to higher linear attenuation coefficients.

## 4. Conclusions

In this paper, nano TiO_2_ powders were initially proposed and added in the silicone-W-based composites to improve the related properties of the composites used in soft nuclear robots. The influence of the addition of nano TiO_2_ powders on the tensile elongation, thermal stability, and gamma-ray-shielding properties of the composites was studied and analyzed for the first time. The conclusions based on our investigation are as follows:

(1) According to the EDS elemental mapping results, the tungsten and nano TiO_2_ powder were observed in uniform dispersion in the silicone matrix.
(2) With the addition of 0.1–2.0 wt.% nano TiO_2_, the total mass losses of the silicone-W-TiO_2_-based composite samples T1, T2, T3, and T4 increased slightly by about 0.80–2.81% compared to that of the silicone-W-based composite sample T0 in the temperature range of 30–800 °C. This demonstrates that the addition of nano TiO_2_ with the weight ratio of 0.1–2.0 wt.% has a slight impact on the thermal stability, compared to that of sample T0 containing 80 wt.% tungsten powders and 20 wt.% silicone.
(3) The optimum improvement in tensile elongation property of the silicone-W-TiO_2_-based composites was achieved with 0.1 wt.% nano TiO_2_ powders, 79.9 wt.% tungsten powders, and 20 wt.% silicone.
(4) The inclusion of 0.1–2.0 wt.% nano TiO_2_ powders resulted in a slight reduction in the gamma-ray linear attenuation coefficient by about 1.1–4.8% and the increase in tensile elongation by about 10.96% at 25 °C, compared with that of sample T0 (80 wt.% tungsten powders and 20 wt.% silicone).

The tensile and gamma-ray-shielding properties are the two main factors that affect the performance of soft robots. Therefore, the research for this purpose comes to a conclusion that the addition of nano TiO_2_ with a certain weight ratio (0.1 wt.% in this paper) has the benefits of enhancing the tensile elongation of the composites by about 10.96% at 25 °C and could also attribute to a slight reduction in the gamma-ray-shielding property, compared to that of the sample made of 80 wt.% tungsten powders and 20 wt.% silicone.

Considering the application in NPPs, a soft nuclear robot may face higher energy gamma rays than those used in these experiments here. The larger energies gamma-ray-shielding property of this flexible composites will be investigated experimentally based on other more-suitable devices in the future.

## Figures and Tables

**Table 1:** Weight fraction of each components and the tensile elongation results of the gamma-ray-shielding composites.

Sample	Silicone (wt.%)	Tungsten (wt.%)	Nano TiO_2_ (wt.%)	Density (g cm^−3^)	Tensile Elongation at 25 °C (%)	Tensile Elongation at 100 °C (%)	Tensile Elongation at 150 °C (%)
T0	20.0	80.0	0	4.513	500.84	724.5	561.97
T1	20.0	79.9	0.1	4.498	555.75	612.34	460.72
T2	20.0	79.5	0.5	4.490	471.24	508.92	380.58
T3	20.0	79.0	1.0	4.455	392.52	414.17	347.05
T4	20.0	78.0	2.0	4.378	264.51	360.65	219.72
T5	100.0	0	0	1.116	715.20	803.00	360.00

**Table 2:** Mass losses of the silicone-W-based composites doped with nano titanium oxide flexible gamma-ray-shielding material samples at different temperature ranges.

Sample	30–200 °C (%)	200–300 °C (%)	300–400 °C (%)	400–500 °C (%)	500–600 °C (%)	600–700 °C (%)	700–800 °C (%)	Total Mass Loss (%)
T0	0.0256	0.1481	0.3559	3.7469	5.7134	1.2906	0.0761	12.3566
T1	0.3269	0.1829	1.8357	9.9686	2.4380	0.0269	0.0031	14.7934
T2	0.7938	0.2863	1.4330	10.7105	1.9411	0.0018	0.0077	15.1661
T3	0.6259	0.3548	1.1297	5.9212	5.0065	0.5564	0.0049	13.5992
T4	0.7879	0.4971	1.2850	3.8897	4.7179	2.2010	0.0193	13.1538

**Table 3:** Attenuation coefficients results of the gamma-ray-shielding composites.

Sample	Silicone (wt.%)	Tungsten (wt.%)	Nano TiO_2_ (wt.%)	Linear Attenuation Coefficient (cm^−1^)	Mass Attenuation Coefficient (cm^2^ g^−1^)	HVL (cm)	MFP (cm)
T0	20.0	80.0	0	0.2064	0.0457	3.3576	4.8450
T1	20.0	79.9	0.1	0.1963	0.0436	3.5429	5.0942
T2	20.0	79.5	0.5	0.1956	0.0435	3.5429	5.1125
T3	20.0	79.0	1.0	0.2034	0.0457	3.4071	4.9164
T4	20.0	78.0	2.0	0.1980	0.0452	3.5000	5.0505
T5	100.0	0	0	0.0517	0.0463	13.4043	19.3424

## Data Availability

All data included in this study are available upon request by contact with the corresponding author.

## References

[1] Haddi Y., Kharchaf A. (2021). Obstacle avoidance behavior of an autonomous mobile robot in a radioactive environment based on fuzzy logic. E3S Web Conf..

[2] Zhang Z., Fu B., Li L., Yang E. (2021). Design and function realization of nuclear power inspection robot system. Robotica.

[3] Tsitsimpelis I., Taylor C.J., Lennox B., Joyce M.J. (2019). A review of ground-based robotic systems for the characterization of nuclear environments. Prog. Nucl. Energy.

[4] Bird B., Griffiths A., Martin H., Codres E., Jones J., Stancu A., Lennox B., Watson S., Poteau X. (2018). A robot to monitor nuclear facilities: Using autonomous radiation-monitoring assistance to reduce risk and cost. IEEE Robot. Autom. Mag..

[5] Kim D., Kim Y.S., Noh K., Jang M., Kim S. (2020). Wall-climbing robot with active sealing for radiation safety of nuclear power plants. Nucl. Sci. Eng..

[6] Sato Y., Tanifuji Y., Terasaka Y., Usami H., Kaburagi M., Torii T. (2019). Radiation imaging using a compact Compton camera mounted on a crawler robot inside reactor buildings of Fukushima Daiichi Nuclear Power Station. J. Nucl. Sci. Technol..

[7] Huang C.-W., Huang C.-H., Hung Y.-H., Chang C.-Y. (2018). Sensing pipes of a nuclear power mechanism using low-cost snake robot. Adv. Mech. Eng..

[8] Sundar G.S., Sivaramakrishnan R., Venugopal S. (2012). Design and developments of inspection robots in nuclear environment: A review. Int. J. Mech. Eng. Rob. Res..

[9] Redus R., Squillante M., Gordon J., Knoll G., Wehe D. (1994). A combined video and gamma ray imaging system for robots in nuclear environments. Nucl. Instrum. Meth. A.

[10] Bakari M.J., Zied K.M., Seward D.W. (2007). Development of a multi-arm mobile robot for nuclear decommissioning tasks. Int. J. Adv. Robot. Syst..

[11] Ducros C., Hauser G., Mahjoubi N., Girones P., Boisset L., Sorin A., Jonquet E., Falciola J.M., Benhamou A. (2017). RICA: A tracked robot for sampling and radiological characterization in the nuclear field. J. Field Robot..

[12] Youn J., Jeong S.M., Hwang G., Kim H., Hyeon K., Park J., Kyung K. (2020). Dielectric elastomer actuator for soft robotics applications and challenges. Appl. Sci..

[13] Szliszka E., Czuba Z.P., Domino M., Mazur B., Zydowicz G., Krol W. (2009). Ethanolic extract of propolis (EEP) enhances the apoptosis-inducing potential of TRAIL in cancer cells. Molecules.

[14] Khanbareh H. (2017). Large area and flexible micro-porous piezoelectric materials for soft robotic skin. Sens. Actuators A Phys..

[15] Li S., Vogt D.M., Rus D., Wood R.J. (2017). Fluid-driven origami-inspired artificial muscles. Proc. Natl. Acad. Sci. USA.

[16] Shimoga G., Choi D.S., Kim S.Y. (2021). Bio-inspired soft robotics: Tunable photo-actuation behavior of azo chromophore containing liquid crystalline elastomers. Appl. Sci..

[17] Held M., Pichler A., Chabeda J., Lam N., Hernandez G. (2021). Soft electronic platforms combining elastomeric stretchability and biodegradability. Adv. Sustain. Syst..

[18] Wang J., Zheng T., Gao Y., Wang D., Wang C. (2020). Preparation and properties characterization of a novel soft robots partially made of silicone/W-based composites for gamma ray shielding. Prog. Nucl. Energy.

[19] Jeong J., Yasir I.B., Han J., Park C.H., Kyung K.U. (2019). Design of shape memory alloy-based soft wearable robot for assisting wrist motion. Appl. Sci..

[20] Allen T., Busby J., Meyer M., Petti D. (2010). Materials challenges for nuclear systems. Mater. Today.

[21] Zinkle S.J. (2013). Materials challenges in nuclear energy. Acta Mater..

[22] Abouhaswa A.S., Abouhaswa A., Zakaly H., Issa S., Ahmed M.R., Yuness M. (2021). Synthesis, physical, optical, mechanical, and radiation attenuation properties of TiO_2_–Na_2_O–Bi_2_O_3_–B_2_O_3_ glasses. Ceram. Int..

[23] Nikbin I.M., Mehdipour S., Dezhampanah S., Mohammadi R., Sadrmomtazi A. (2020). Effect of high temperature on mechanical and gamma ray shielding properties of concrete containing nano-TiO_2_. Radiat. Phys. Chem..

[24] Kilicoglu O. (2020). Bioactive glasses with TiO_2_ additive: Behavior characterization against nuclear radiation and determination of buildup factors. Ceram. Int..

[25] Al-Hadeethi Y., Sayyed M.I., Al-Buriahi M.S. (2020). Bioactive glasses doped with TiO_2_ and their potential use in radiation shielding applications. Ceram. Int..

[26] Ndong R.S., Russel W.B. (2011). Effects of molecular weight and volume fraction on rheological properties of PDMS-grafted alumina in PDMS melts. J. Rheol..

[27] Yao Y., Lu G.Q., Boroyevich D., Ngo K. (2014). Effect of Al_2_O_3_ fibers on the high-temperature stability of silicone elastomer. Polymer.

[28] Ryu T., Hwang K.S., Choi Y.J., Hong Y.S. (2009). The sintering behavior of nanosized tungsten powder prepared by a plasma process. Int. J. Refract. Met. Hard Mater..

[29] Ryu T., Sohn H.Y., Hwang K.S., Fang Z.Z. (2009). Chemical vapor synthesis (CVS) of tungsten nanopowder in a thermal plasma reactor. Int. J. Refract. Met. Hard Mater..

[30] Zhang D., Cai Q., Liu J. (2012). Formation of nanocrystalline tungsten by selective laser melting of tungsten powder. Mater. Manuf. Process..

[31] Almurayshid M., Alssalim Y., Aksouh F., Almsalam R., ALQahtani M., Sayyed M.I., Almasoud F. (2021). Development of new lead-free composite materials as potential radiation shields. Materials.

[32] Mheemeed A., Hasan H., Al-Jomaily F. (2012). Gamma-ray absorption using rubber—lead mixtures as radiation protection shields. J. Radioanal. Nucl. Chem..

